# Revolutionizing Medicine: Chatbots as Catalysts for Improved Diagnosis, Treatment, and Patient Support

**DOI:** 10.7759/cureus.80935

**Published:** 2025-03-21

**Authors:** Syed A Abbas, Izmir Yusifzada, Sufia Athar

**Affiliations:** 1 Health, Delhi Public School - Modern Indian School, Doha, QAT; 2 Health, Secondary School Number 210, Baku, AZE; 3 Department of Obstetrics and Gynaecology, Al Wakra Hospital, Hamad Medical Corporation, Doha, QAT

**Keywords:** artificial intelligence, chatbot, health, prevention, screening

## Abstract

Chatbots have emerged as one of the revolutionary tools in healthcare that combine artificial intelligence to improve patient engagement in diagnosis, treatment, monitoring, and support. In this review, the application of chatbots in medicine over the past two decades is discussed, focusing on their application in several aspects of medicine, including oncology, psychiatry, and chronic disease management. A total of 38 clinically relevant studies were identified from a comprehensive literature search. These studies demonstrate that the efficacy of chatbots in early symptom detection, personalized treatment planning, and emotional support has a particular emphasis on addressing mental health needs. The review focuses on the role of chatbots in improving the communication gap between patients and health providers to achieve improved access to medical information and treatment adherence. The findings underscore the substantial promise of chatbots in enhancing healthcare delivery and reveal areas that need further exploration, particularly in radiology and advanced diagnostics. With the continuous rise in technological development, the use of chatbots is expected to become more central in streamlining health processes toward better patient outcomes and enhanced health literacy. This review, therefore, calls for continued investment in the incorporation of these chatbot innovations in healthcare systems for their full potential to be realized in patient care.

## Introduction and background

Since the development of modern technologies, chatbots have found implementation in different spheres of life. Equipped with software capable of simulating human communication, chatbots quickly respond to modern needs for flexibility and an integrated approach to conversation. Recently, the use of chatbots in the medical and healthcare industries has been amplified. The possibility of chatbots within the medical context is enormous, starting from the initial consultation showing a sense of support to the patients to providing diagnosis and the necessary information concerning the treatment process. An individual approach to patients and the processing of huge volumes open new perspectives in improving medical care and adjusting healthcare processes, respectively [1].

Over the past two decades, research has made notable strides in elucidating the utility of various chatbot applications in areas such as patient assistance, health promotion, diagnosis, and treatment. The use of chatbots in medical practice now gives a basis for continued investigation into their future possibilities [1,2].

Present findings establish that the successful integration of chatbots is already in place for most medical applications. For instance, the use of chatbots for diagnosis in the issuance of necessary treatment recommendations and in the detection of symptoms in their early stages [3-5]. On the other hand, and equally important, other fields where the presence of a chatbot has been strongly felt are within the scope of mental health. Examples of successful applications of chatbots in the surveillance and support of mental health include resources and recommendations on symptom management [5-7]. The huge potential of using chatbots for timely information on vaccinations underlines their importance in tackling global health crises [8]. Further, chatbots do not even have to bear the risk of engendering nominal performances in the treatment process but can instead support diagnoses efficiently and effectively to guarantee quicker and more accurate reporting in medical contexts. They can also help, to some extent, in the treatment planning and management of common diseases with minimal need for an immediate consultation with a doctor. Chatbot integration in healthcare can improve patient support services, particularly for those with chronic illnesses such as cancer. It would be quite beneficial to use them to promote health-related activities, such as immunization and mental wellness, particularly during a public health emergency [3-7].

Despite all of these advantages, there are several difficulties with deploying chatbots in healthcare and medicine. First, several concerns revolve around the necessity for high dependability and accuracy in the data used by chatbots to make choices. A solution to this problem will help prevent the dissemination of erroneous medical information that might lead to serious health implications for patients. Managing intricate medical conversations and acknowledging patients’ emotional and psychological needs when interacting with chatbots are further challenges. This drawback suggests that further research and development are required before they can be incorporated into electronic medical records and other systems in healthcare facilities [9]. This narrative review assesses the latest developments and uses of chatbots in medicine, highlighting their contributions to patient care, diagnosis, therapy, and health promotion, especially in the field of public health.

## Review

Methodology

This narrative review aimed to comprehensively assess the current status of chatbot technology in healthcare. A systematic searching strategy was applied in several databases, including PubMed, Google Scholar, and Web of Science. The search utilized a list of precise keywords, such as “chatbot technology,” “healthcare,” “diagnosis,” “patient support,” “treatment planning,” and “health promotion,” with Boolean operators (AND, OR) applied to refine the results. Inclusion criteria were tailored to restrict the literature search to clinical research articles published within the last 20 years which specifically examined chatbot applications in diagnosis, treatment, patient care, and health promotion. Screening involved an initial title and abstract screening, followed by full-text scrutiny of potentially valid articles to check if they fulfilled the inclusion criteria. The selection process aimed to attain a balanced combination of studies to address relevant and significant findings. To check the reliability and validity of the studies included, a comprehensive evaluation of bias was conducted using a systematic quality screening process. Each of the 36 included articles were assessed for methodological quality, appropriateness of sample size, and presentation of results, utilizing standardized instruments such as the Cochrane Risk of Bias tool for randomized trials and the Risk of Bias in Non-randomized Studies of Interventions instrument for non-randomized trials. Some items evaluated were potential selection bias in participant recruitment, performance bias in the care process, detection bias in measuring outcomes, and attrition bias due to dropouts or missing data. This thorough review was performed by independent reviewers to minimize subjective interpretation and enhance objectivity to reach a consensus on the quality of evidence. An overview of the chosen articles was part of the data extraction and analysis process, which also included classifying and combining the results to show the variety of chatbot applications. In particular, this research assessed chatbots’ effectiveness in terms of speed and accuracy of diagnosis, clarified their functions in treatment planning and patient care, and examined the results of chatbot interventions regarding patient support and health promotion. The review also discussed the drawbacks and difficulties with chatbot use that have been noted in the literature.

Role of chatbots in diagnosis

Among the diagnostic chatbots, five studies were included. It was noted that the screening applications are outstanding for assessing hereditary cancer risks based on family history by the chatbots IBM Watson and ItRuns [3-5]. The keen interest in this area by several researchers illustrates the potential of artificial intelligence (AI) for the early identification of populations at risk in specialties such as psychiatry, oncology, and cardiology.

ItRuns is an eHealth platform for the enhancement of patient participation in health management and simplification of integrated healthcare processes, particularly regarding oncologic screening. With support provided to the learning processes about cancer risk and its screening, ItRuns motivates patients to be more active. It allows for the collection and management of important information at the patient level, such as family history and symptoms described by the patients themselves, which is significant during the assessment of cancer risk. It enables healthcare professionals to more actively observe changes in their patients’ health through remote monitoring and provides opportunities for the early detection of possible cancer concerns. It streamlines specialist referrals for further screenings and integrates with electronic health records (EHRs) for improved access to patient data, further enhancing the coordination of care and early detection even [3].

IBM Watson is an advanced, AI platform that uses natural language processing and machine learning for evidence-based decision-making in tumors. It systematically analyzes data, including EHRs and clinical research, to help healthcare professionals make the right decisions on the medical tests to recommend for patients according to their risk profile. Watson enhances the risk level of genetic conditions, lifestyle choices, and family history, providing higher levels of personalization in cancer prevention. Equipped with rapid synthesis and analysis capabilities, it aids in the elaboration of patterns which would provide the necessary information on the adoption of screening protocols to be assured of timely referrals for specialist testing. By integrating easily into the workflow, IBM Watson’s data-driven insights help clinicians improve the quality and efficiency of cancer screening practices [5].

Table 1 gives an overview of the types of chatbot applications in the different fields of medicine, specific functions, areas of application, and the number of studies conducted within each category. An analysis of the data points yields information on the role and effectiveness of chatbots in the improvement of healthcare delivery.

**Table 1:** Chatbot applications in healthcare.

Chatbot application	Application/Chatbot	Field of application	Number of studies (n)
Screening	ItRuns [3], IBM Watson [4,5]	Psychiatry, Oncology, Cardiology	3
Symptom detection	MindLAMP [6], BiAffect [7,9,10], CoV-19 Chatbot [8], Cardiobot [11], Urochat [12], KakaoTalk [13], Ada [14], DoctorBot [15], MP15-03 [16], Arogi [17], Medical Sieve [18]	Psychiatry, Oncology, Medicine, Cardiology, Urology, Obstetrics and Gynecology, SARS-CoV-2	12
Radiodiagnostic	Medical Sieve [18]	Oncology	1
Treatment	Watson for Oncology [19], Madhu, Mathew [20,21]	Oncology, Medicine, Psychiatry	3
Monitoring	AiCure [22], Memora Health [23], STREAMD [24], Conversa [25]	Medicine, Oncology, Psychiatry	4

Chatbots for the detection of symptoms

The preceding section addressed the role of chatbots in diagnosis, this section focuses on how chatbots facilitate symptom detection by providing users with personalized assessments, real-time feedback, and actionable insights tailored to various medical domains. Chatbots, including mindLAMP, BiAffect, Cardiobot, counUrochat, KakaoTalk, Ada, DoctorBot, MP15-03, and Arogi Chatbot, demonstrate the development in the recent surge toward the application of AI and digital health technology in early symptom diagnosis in several medical areas. Each tool has different domains in which it functions optimally to meet users’ needs effectively.

Chatbots have become increasingly popular in the healthcare industry, proving their adaptability to a wide range of medical specialties. Tools such as mindLAMP (which stands for “Learn, Assess, Manage, Predict”) use real-time data to track a person’s emotional and psychological states in the field of psychiatry. MindLAMP can identify disorders such as anxiety and depression by facilitating frequent check-ins and performing symptom evaluations. This creative solution also helps people control their mental health by guiding them through self-management techniques and suggesting interventions specific to their requirements [6]. The development of chatbot technology has also become crucial in the field of infectious diseases, particularly considering public health hazards such as SARS-CoV-2. The need for efficient communication and information sharing has been highlighted by the increased knowledge of quickly changing diseases, making chatbots an invaluable tool in handling these public health issues [8].

Chatbots such as BiAffect are used in oncology to help with early cancer symptom detection, highlighting the urgent need for prompt interventions. These chatbots seek to enhance outcomes by enabling early detection of possible cancerous indications and facilitating timely follow-ups and medical consultations [7,9,10]. Advanced chatbots such as CardioBot, which specializes in heart health management, are beneficial in cardiology. CardioBot helps users evaluate their cardiovascular symptoms, finds risk factors, and suggests lifestyle modifications or required medical appointments. Additionally, it helps individuals communicate their health information and symptoms to medical experts in an efficient manner, which improves their quality of care [11].

For people looking for information on urological diseases and treatments, UroChat is a specialized resource in the field of urology. The chatbot simplifies the process of accessing care by assessing symptoms and referring users to the right medical specialists or resources depending on the difficulties they report [12]. Similarly, chatbots such as KakaoTalk have been developed to treat women’s health issues in the field of obstetrics and gynecology. To assist users in navigating reproductive health difficulties, they offer symptom checks with customized settings and tracking, as well as recommendations for healthcare services [13].

Chatbot technology is also used in general medicine and specialist care. Programs such as Ada provide thorough health examinations and triage solutions for a variety of ailments. A conversational symptom checker offered by Buoy Health helps users explain their symptoms and then makes recommendations for possible diagnosis and the next stages in treatment [14]. By helping users with health-related questions and navigating healthcare services, the AI-powered DoctorBot improves patient engagement and supports information management and follow-up care [15].

Despite being less well-known, the chatbot MP15-03 is useful in clinical settings, especially in managing chronic disease treatment planning. It encourages patient empowerment and involvement by providing feedback depending on user inputs [16]. By using AI-driven dialogues to provide symptom assessments and direct patients toward the right healthcare resources, Arogi tackles the difficulties associated with healthcare delivery in regions where access to medical professionals is restricted [17]. Finally, Medical Sieve helps radiologists make correct diagnoses from medical imaging by serving as a clinical decision support system. It reduces diagnostic errors and enhances training for both novice and seasoned practitioners by allowing users to enter clinical symptoms or imaging results, which are then used to create differential diagnoses based on a large database [18].

The implementation of chatbots in a variety of medical fields represents an encouraging milestone in the provision of healthcare, providing individualized support, increasing the precision of diagnoses, and expediting patient communication with healthcare systems through dialogues powered by AI. This capacity is particularly important for underprivileged groups, as it enhances access to healthcare and education.

Chatbots in treatment

After a diagnosis is established, chatbots can augment the process by using symptom-checking algorithms and patient-reported data to provide preliminary diagnoses and recommendations. Such early integration aids in overcoming delays between patients’ initial presentation and formal medical evaluation, reducing delays in rescheduled or missed appointments. Interestingly, the domain of treatment shows the most interest in using chatbots for symptom assessment and therapy suggestions [12,19-21]. Chatbots with predictive analytics can assist in the personalization of care, particularly in oncology, medicine, and psychiatry, highlighting the transformative potential of these tools in these fields. Six of the studies used application monitoring solutions such as AiCure and Memora Health. These examples highlight a chatbot’s integrated role in patient engagement and education, which is especially important in chronic disease management.

IBM Watson analyzes large volumes of medical literature, patient records, and clinical trial data that help make informed decisions regarding treatments. It helps healthcare providers make evidence-based decisions in presenting appropriate choices of therapy or treatment for specific conditions. It provides information on patients’ medical history and genetic data to help generate personalized treatment plans. This is particularly useful in oncology, where personalized medicine plays a crucial role. It aids in clinical decision support for care providers in providing real-time insights and evidence-based recommendations for treatments to clinicians at the point of contact with a patient. This serves to raise the quality of outcomes [19]. Madhu Chatbot can educate patients regarding their medical condition and the treatment prescribed, likely improving understanding and adherence to the treatment plan. It regularly monitors the symptoms and any side effects experienced by the patients to identify any complications that may arise and make changes in treatment. It can assist in scheduling follow-up appointments and even sends reminders to patients for tests or consultations that they need to take, thus assuring continuity of care [12]. Chatbot Mathew is often used in managing chronic conditions by providing resources for self-management to the patient and encouraging treatment adherence through reminders and motivational support. It can help the patient remember and monitor prescribed drugs. This includes dosage, time, and all side effects or interactions the drugs taken may have for adherence to treatment programs. Patients are counseled and supported by different lifestyle changes necessary for effective treatment, such as dietary modifications or exercises prescribed for conditions such as diabetes or hypertension [20,21]. There are many chatbots in complementary but different roles in treatment. They promote better patient care by enhancing the efficiency, efficacy, and personalization of healthcare treatment strategies.

Chatbots for patient monitoring

Continuous monitoring is crucial in individuals with chronic illnesses to prevent the repercussions of uncontrolled health status. Various chatbots such as AiCure, Memora Health, STREAMD, and Conversa are in use for monitoring patients. AiCure has AI and computer vision for real-time monitoring of patient adherence to medication. It confirms using video feeds that patients are correctly taking their medications. This tool can track patient behaviors indicative of their medication and health management to assist healthcare providers in the identification of patients who could be at risk for non-adherence. The application provides collected data on patient interactions to clinicians, informing them regarding adherence patterns and barriers to treatment [22]. Memora Health uses a chatbot to conduct automated check-ins through dialogue to monitor patient symptoms and health status. It creates patient-centric care pathways, reminders, and data and response collection to modify any care plans for continuous monitoring. It consolidates data gathered from patient conversations and presents healthcare providers with actionable insights to help them make decisions on follow-up treatments [23].

STREAMD monitors patients suffering from chronic ailments by enabling them to track their symptoms and health data using a conversational interface. Patients can report any changes in their health status, which health professionals can observe and respond to when necessary. The platform can allow for communication between the patients and their healthcare teams, making the coordination of care easier and enabling appropriate alterations in treatment plans with increased timeliness [24]. Conversa increases patient engagement through regular communication by sending reminders and health check-ins that prompt patients to improve their health status. With conversational AI, Conversa allows real-time data to be captured on symptoms, mood, and adherence to therapies that will enhance the proactive management of patient health. It allows the integration of data into EHRs so that healthcare providers can have a clear idea of the health status of a patient over time, helping them make more informed decisions [25]. Such chatbots in patient monitoring support patients continuously, facilitate compliance with medication, allow for automated check-ins, and enable patients to report their current health status. This helps in enhancing healthcare delivery efficiency, improves patient adherence and active engagement, and has valuable input for clinicians for effective care management. As these technologies evolve, their roles in monitoring patient health are also expected to broaden toward improved health outcomes.

Chatbots in patient support

Monitoring alone is insufficient for individuals with chronic illnesses, who require assistance throughout their treatment journey. Some chatbots have been integrated into patient care to provide support, notably to those with mental health issues. Chatbots such as Unmind and Replika are used to provide emotional support and psychoeducation for psychiatry and pediatrics. This suggests a recognition that mental health and emotional well-being are now an essential part of whole-person care. Health promotion, including the use of Chatbots such as SWITCHes and CoachA for healthy lifestyle choices, has been explored. This trend implies that there is an increasing orientation toward prevention and health education, which are very crucial for the prevention of chronic diseases and for ensuring public health. Chatbots, such as Unmind, Replika, Shim, Tess, and Vik, are used to offer support to patients. Unmind is an online mental health platform built to boost workplace well-being. It has many tools and resources that can help deal with stress, anxiety, and other mental health concerns. The chatbot from Unmind can interact with users by offering them more personalized content, assessing their mental health, and guiding them toward a coping strategy for improved mental well-being [26].

Replika is an AI-driven companion conversational agent designed for emotional support and companionship. Users can share feelings or experiences with Replika, chat about anything, or even talk about unimportant issues. It reduces loneliness and anxiety by creating an environment where users can express themselves with no restraint and receive empathetic responses in return [27]. Shim is a chatbot focused on men’s health, especially sexual and reproductive health. It provides users with a safe and anonymous environment to ask questions, understand symptoms, and find health knowledge of concern to them. By providing correct information, reducing stigma, and encouraging proactive health management, Shim hopes to empower the user [28].

Tess is an AI-enabled mental health chatbot providing real-time emotional support through texting. Users can express their emotions with Tess. The chatbot, in turn, implements coping strategies, mindfulness exercises, and referrals to mental health services when needed. This constant companion is always available to help steer the user through difficult emotions and situations [29]. The emphasis of Vik is on chronic disease patients. It engages the user in personal conversations regarding the monitoring of symptoms, and it reminds users to take their medicines on time. Vik encourages treatment plan adherence and can assure them that they are connected and supported in the management of health, thus enabling care and self-management [30]. These chatbots also provide crucial support for patients in terms of emotional support, health information, and personalized contact. They fill gaps in care, decrease the burden of communication barriers, and improve outcomes for mental and physical health [31-33].

Chatbots in health promotion

Chatbots in health promotion can be seen as a tool of interaction that may enhance patient engagement through improved education and tracking of behaviors. By facilitating accessible real-time communication, these AI-driven platforms facilitate dialogue and provide personalized health information, reminding patients about medication and appointment adherence. They can gather valuable data on patient behaviors and experiences that enable healthcare providers to identify trends in patterns to help improve interventions. Chatbots can also provide personalized lifestyle advice and apply cognitive behavioral skills to further enhance health outcomes. Generally, integration into health systems enables them to be available for the patients always, while barriers to access and health literacy are being minimized [34].

Paola is a virtual health coach that leads users through personalized wellness plans, focusing on setting and achieving health goals, motivational support, and customizing exercise and nutrition programs. Through continuous feedback and periodic engagement, CoachA plays a major role in promoting physical activity and overall health literacy [35].

WeightMentor focuses on weight management and prevention of obesity. This conversing chatbot facilitates users to monitor their food intake and physical activities against weight changes. It also provides educational resources about nutrition, portion control, and healthy eating to support users in making informed choices for sustainable weight loss or maintenance [36].

Health Hero aims to improve overall wellness by highlighting healthier life habits to users. Most have gamification features, which health programs entertaining to go through. This allows users to receive rewards when they reach certain health milestones or complete wellness challenges. Through this level of interactivity, one can create community support among users while boosting physical health and emotional well-being [37].

Cory COVID-Bot is an AI-powered chatbot collaboratively developed across sectors to support people in safely behaving toward a “COVID-19 normal” life. It addresses the challenge of effectively imparting COVID-19-safe behaviors among young, culturally and linguistically diverse populations. Most notably available in both English and Vietnamese, Cory COVID-Bot effectively engages hard-to-reach communities with its clear, updated public health recommendations. Its main tasks include decreasing public confusion regarding health directions and incentivizing safe behavior with evidence-based behavior change communication to limit, at the minimum, the occurrence of unnecessary harm and illness due to the pandemic [38].

Each chatbot plays a different role in health promotion, targeting different dimensions of wellness. They provide personalized support, education, and motivation that enable users to change toward healthier behaviors and achieve health goals. These chatbots leverage technology in making engagement easy and tracking progress to further an individual’s endeavor for health and well-being.

## Conclusions

The use of different chatbot applications in healthcare is promising for the betterment of patient engagement in diagnosis, treatment, monitoring, and support. Various medical fields are finding chatbot applications to be an important tool in the early detection of symptoms by providing choices for personalized treatment planning, especially in oncology, psychiatry, and management of chronic diseases. The increasing number of studies underlines an increasingly greater recognition of the roles chatbots play in improving healthcare delivery and personalizing patient care through predictive analytics and effective communication. Active chatbots for monitoring and emotional support prove effective in enhancing treatment adherence and management of the mental health status of patients, thus contributing to holistic healthcare. The use of such platforms reduces communication gaps not only between a patient and healthcare provider but also empowers the patient through personalized advice, tracking health behaviors, and facilitating easier access to medical information. Considering the applications that have been developed and are proving promising, there is a need for further research, especially in unexploited areas of radiology and specialized diagnostics, for maximum contribution from chatbots in clinical settings. It is expected that as technology evolves, so will the functionality of chatbots, invariably increasing their effectiveness in promoting health among patients, streamlining healthcare processes, and responding to public health challenges. Sustained investment in chatbot innovation and integration into healthcare systems will realize the potential for improved patient outcomes and overall health literacy.

## References

[1] Adamopoulou E, Moussiades L (2020). Chatbots: history, technology, and applications. Mach Learn Appl.

[2] Wilson L, Marasoiu M (2022). The development and use of chatbots in public health: scoping review. JMIR Hum Factors.

[3] Allen C (2023). User experience of a family health history chatbot: a quantitative analysis. Research Square.

[4] Johnson KB, Wei WQ, Weeraratne D (2021). Precision medicine, AI, and the future of personalized health care. Clin Transl Sci.

[5] Langholm C, Breitinger S, Gray L (2023). Classifying and clustering mood disorder patients using smartphone data from a feasibility study. NPJ Digit Med.

[6] Vaidyam A, Halamka J, Torous J (2022). Enabling research and clinical use of patient-generated health data (the mindLAMP platform): digital phenotyping study. JMIR Mhealth Uhealth.

[7] Vesel C, Rashidisabet H, Zulueta J (2020). Effects of mood and aging on keystroke dynamics metadata and their diurnal patterns in a large open-science sample: a BiAffect iOS study. J Am Med Inform Assoc.

[8] Nam T (2020). How did Korea use technologies to manage the COVID-19 crisis? A country report. Int Rev Public Adm.

[9] Blandford A, Gibbs J, Newhouse N, Perski O, Singh A, Murray E (2018). Seven lessons for interdisciplinary research on interactive digital health interventions. Digit Health.

[10] Ross MK, Tulabandhula T, Bennett CC (2023). A novel approach to clustering accelerometer data for application in passive predictions of changes in depression severity. Sensors (Basel).

[11] Yılmaz A (2023). Deep Learning and Cardiology: Revolutionizing Diagnosis, Management, and Prognosis in Cardiovascular Medicine. CardioBot: Harnessing Deep Learning for Groundbreaking Innovations in Cardiac Care.

[12] Talyshinskii A, Naik N, Hameed BM (2023). Expanding horizons and navigating challenges for enhanced clinical workflows: ChatGPT in urology. Front Surg.

[13] Chung K, Cho HY, Park JY (2021). A chatbot for perinatal women's and partners' obstetric and mental health care: development and usability evaluation study. JMIR Med Inform.

[14] Jungmann SM, Klan T, Kuhn S, Jungmann F (2019). Accuracy of a chatbot (Ada) in the diagnosis of mental disorders: comparative case study with lay and expert users. JMIR Form Res.

[15] Khaire PS, Shahane V, Borse P, Jundhare A, Tatu A (2022). Doctor-Bot: AI powered conversational chatbot for delivering e-health. Int J Res Appl Sci Eng Technol.

[16] Kobori Y, Osaka A, Soh S, Okada H (2018). MP15- 03: Novel application for sexual transmitted infection screening with an AI chatbot. J Urol.

[17] Sharma D, Kumar H, Kaushal S, Gainder S (2023). Arogi Chatbot: a platform for pre-screening gynecology and obstetrics patients. IC3-2023: Proceedings of the 2023 Fifteenth International Conference on Contemporary Computing.

[18] Syeda-Mahmood T, Walach E, Beymer D (2016). Medical sieve: a cognitive assistant for radiologists and cardiologists. Med Imaging.

[19] Xu L, Sanders L, Li K, Chow JC (2021). Chatbot for health care and oncology applications using artificial intelligence and machine learning: systematic review. JMIR Cancer.

[20] Pham KT, Nabizadeh A, Selek S (2022). Artificial intelligence and chatbots in psychiatry. Psychiatr Q.

[21] Lim SM, Shiau CW, Cheng LJ, Lau Y (2022). Chatbot-delivered psychotherapy for adults with depressive and anxiety symptoms: a systematic review and meta-regression. Behav Ther.

[22] Salcedo J, Rosales M, Kim JS, Nuno D, Suen SC, Chang AH (2021). Cost-effectiveness of artificial intelligence monitoring for active tuberculosis treatment: a modeling study. PLoS One.

[23] Lau-Min KS, Marini J, Shah NK (2024). Pilot study of a mobile phone chatbot for medication adherence and toxicity management among patients with GI cancers on capecitabine. JCO Oncol Pract.

[24] Campbell K, Louie P, Levine B, Gililland J (2020). Using patient engagement platforms in the postoperative management of patients. Curr Rev Musculoskelet Med.

[25] Dinh-Le C, Chuang R, Chokshi S, Mann D (2019). Wearable health technology and electronic health record integration: scoping review and future directions. JMIR Mhealth Uhealth.

[26] Sierk A, Travers E, Economides M, Loe BS, Sun L, Bolton H (2022). A new digital assessment of mental health and well-being in the workplace: development and validation of the Unmind Index. JMIR Ment Health.

[27] Ma Z, Mei Y, Su Z (2024). Understanding the benefits and challenges of using large language model-based conversational agents for mental well-being support. AMIA Annu Symp Proc.

[28] Baradaran N, Awad M, Gaither TW (2019). The association of bicycle-related genital numbness and Sexual Health Inventory for Men (SHIM) score: results from a large, multinational, cross-sectional study. BJU Int.

[29] Chaix B, Bibault JE, Pienkowski A, Delamon G, Guillemassé A, Nectoux P, Brouard B (2019). When chatbots meet patients: one-year prospective study of conversations between patients with breast cancer and a chatbot. JMIR Cancer.

[30] Suehs CM, Vachier I, Galeazzi D, Vaast F, Cardon F, Molinari N, Bourdin A (2023). Standard patient training versus Vik-Asthme chatbot-guided training: 'AsthmaTrain' - a protocol for a randomised controlled trial for patients with asthma. BMJ Open.

[31] Fulmer R, Joerin A, Gentile B, Lakerink L, Rauws M (2018). Using psychological artificial intelligence (Tess) to relieve symptoms of depression and anxiety: randomized controlled trial. JMIR Ment Health.

[32] Roca S, Sancho J, García J, Alesanco Á (2020). Microservice chatbot architecture for chronic patient support. J Biomed Inform.

[33] Barwick M, Barac R, Kimber M (2020). Advancing implementation frameworks with a mixed methods case study in child behavioral health. Transl Behav Med.

[34] Singh B, Olds T, Brinsley J (2023). Systematic review and meta-analysis of the effectiveness of chatbots on lifestyle behaviours. NPJ Digit Med.

[35] Maher CA, Davis CR, Curtis RG, Short CE, Murphy KJ (2020). A physical activity and diet program delivered by artificially intelligent virtual health coach: proof-of-concept study. JMIR Mhealth Uhealth.

[36] Holmes S, Moorhead A, Bond R, Zheng H, Coates V, McTear M (2018). WeightMentor: a new automated chatbot for weight loss maintenance. In. Proceedings of the 32nd International BCS Human Computer Interaction Conference. BCS Learning & Development.

[37] Bays DK, Verble C, Powers Verble K (2024). A brief review of the efficacy in artificial intelligence and chatbot-generated personalized fitness regimens. Strength Cond J.

[38] Van Baal ST, Le S, Fatehi F, Hohwy J, Verdejo-Garcia A (2022). Cory COVID-Bot: an evidence-based behavior change chatbot for COVID-19. Stud Health Technol Inform.

